# Assessment of the Quality of Multiple-Choice Questions in the Surgery Course for an Integrated Curriculum, University of Bisha College of Medicine, Saudi Arabia

**DOI:** 10.7759/cureus.50441

**Published:** 2023-12-13

**Authors:** Ahmed Y Al Ameer

**Affiliations:** 1 Department of Surgery, College of Medicine, University of Bisha, Bisha, SAU

**Keywords:** saudi arabia, university of bisha, surgery, psychometric analysis, mcq

## Abstract

Introduction: Multiple-choice questions (MCQs) have been recognized as reliable assessment tools, and incorporating clinical scenarios in MCQ stems has enhanced their effectiveness in evaluating knowledge and understanding. Item analysis is used to assess the reliability and consistency of MCQs, indicating their suitability as an assessment tool. This study aims to ensure the competence of graduates in serving the community and establish an examination bank for the surgery course.

Objective: This study aims to assess the quality and acceptability of MCQs in the surgery course at the University of Bisha College of Medicine (UBCOM).

Methods: A psychometric study evaluated the quality of MCQs used in surgery examinations from 2019 to 2023 at UBCOM in Saudi Arabia. The MCQs/items were analyzed and categorized for their difficulty index (DIF), discrimination index (DI), and distracter efficiency (DE) Fifth-year MBBS students undergo a rotation in the department and are assessed at the end of 12 weeks. The assessment includes 60 MCQs/items and written items. Data was collected and analyzed using SPSS version 24.

Results: A total of 189 students were examined across five test sessions, with 300 MCQ items. Student scores ranged from 28.33% to 90.0%, with an average score of 64.6%±4.35. The 300 MCQ items had a total of 900 distractors. The DIF was 75.3% for the items, and 63.3% of the items showed good discrimination. No items had negative points in terms of biserial correlation. The mean number of functional distractors per test item was 2.19±1.007, with 34% of the items having three functional distractors.

Conclusion: The psychometric indices used to evaluate the MCQs in this study were encouraging, with acceptable DIF, distractor efficiencies, and item reliability. Providing robust faculty training and capacity-building is recommended to enhance item development skills.

## Introduction

Surgical tutors face many difficulties in assessing medical students’ achievements in surgery and using multiple-choice questions (MCQs) requires scrutiny of item quality. Incorporating MCQs by the United States National Board of Medical Examiners in 1978 as an assessment tool in medical testing helps surgical tutors assess medical students’ surgical cognitive skills [1]. The evolution of MCQs continued, becoming the most common and reliable assessment tool [2].

Introducing clinical scenarios into MCQs has improved the assessment of relevant knowledge and understanding and has helped to assess critical thinking, ethics, and professionalism [3,4]. The MCQs are assigned a cognitive level based on the level of Bloom’s taxonomy of specific learning outcomes in the curriculum [3]. Hence, the construction of MCQs depends on the curriculum and must be comprehensive to ensure their applicability to assess the students’ cognitive skills. The medical students’ cognitive skills may cost a patient’s life in the future, so high-quality assessment tools must be used. The critical analysis of surgical MCQs should improve the quality of testing and should also help to drive the learning process [5]. The questions, as assessment tools, must meet specific criteria, but some of these criteria can only be measured after use to ensure high quality.

The psychometric analysis of the MCQs is defined because of events to collect data from MCQs retrospect to their usage as an assessment tool to assess their quality according to specific values [5]. One of the essential aspects of item analysis of the MCQs is to know the reliability or consistency [6]. The overall reliability of a test indicates consistency, homogeneity, and acceptability as an assessment tool. On the other hand, the validity of the test is the ability of a test to measure accurately what it is supposed to measure. Evidence shows that validity is affected by many factors such as quality of test items, number of test items, qualification of item writing skills, and psychometric characteristics of items [4]. The language background of the students has been mentioned as an essential factor by some authors [5].

The estimation of the reliability of the entire test is normally given on the test analysis printout and is usually measured by Kuder-Richardson-20 (KR-20). In comparison, the reliability of individual items is measured by the point-biserial coefficient. The reliability reflects the extent to which the test would give the examiners the same rank if re-admitted without effect to the first administration. Reliability (R) of 0.7-1.0 is considered an acceptable range depending on the type of test, e.g., 0.8 is acceptable for end-course examinations while 0.7 is for other assessments that are not core course examinations like general studies [4,5,7].

The difficulty of the item, in item analysis, is its ability to distinguish between students who know and those who do not [4,6]. It measures the percentage of students who answered the question correctly and ranges between 0 and 1 [8,9]. The difficulty index (DIF I) of an easy question is near 1, while the problematic question is near 0; the desirable range is 0.3-0.7 [9]. The item discrimination index (DI) measures the ability of the item to distinguish between high-performance and low-performance students. It ranges from -1 to +1; negative DI indicates that the number of low-performance examinees correctly answer the item. An item with a DI of 0.2 or less is poor and should be removed [10,11].

Logically, the item of high DIF should have better discrimination, but only sometimes [12]. The distractors are the alternative answers to the correct answer. The efficiency of distractors in MCQ with three distractors is 99.9% if all three distractors are perfectly working, 66.6% if two distractors are working, and 33.3% if only one distractor is working. A functional distractor is the distracter chosen by 5% or more of the examinees [13]. The construction of MCQs with functional distractors is complex and might play an essential role in DI [14,15]. The objective was to determine the quality of MCQs in the surgery course at the University of Bisha College of Medicine (UBCOM) and its acceptability as an assessment tool. The research question: Is the quality of the MCQs acceptable across five years of assessment? Null hypothesis: There is no significant difference in the quality of MCQs in surgery based on psychometric analysis. Alternative hypothesis: There is a significant difference in the quality of MCQs in surgery based on psychometric analysis?

## Materials and methods

Background

The study was conducted at the Department of Surgery, College of Medicine, University of Bisha. The college was established in 2015 and has adopted an integrated SPICES curriculum for MBBS training. The college has about 17 departments, including surgery. The assessment is based on the curricular contents, and MCQs are one of the methods of assessment in surgery.

Study Design

It is a retrospective secondary data review of surgery examinations over five consecutive years. Item analysis was reviewed and used in developing the manuscript.

Study Population

It is an exit examination of 189 candidates conducted from 2019 to 2023. No human subjects were used for this study.

Methods

A psychometric study assessed the quality of MCQs for the surgery examinations administered from 2019 to 2023 at the College of Medicine University of Bisha, Saudi Arabia. The study was conducted at the Department of Surgery over five consecutive years. In each session, a group of fifth-year MBBS students undergo a rotation in the department and get assessed at the end of 12 weeks.

A summative assessment consisting of 60 MCQs/items and written items was administered as part of the evaluation to determine the level of knowledge, skills, and competencies gained. The MCQ items consist of the main stem, four options made up of the key, and three distractors. It is a single best type of question, and students are asked to select the best answers from the four given options. For every correct response, a score of “1” was given, and “0” for incorrect or no response, and negative markings were never used in conducting the assessments. An item analysis was done to validate each item based on each student's performance on the item.

Data collection and analysis

The MCQs/items were analyzed and categorized for their DIF, DI, and distracter efficiency (DE). The internal reliability value of each test score was obtained using the Kuder-Richardson index (KR-20). The results were presented based on the reliability and quality of the test items. Simple proportions, mean, standard deviations, correlation statistics, and t-tests were used to analyze and determine the discrimination and DIFs, respectively. The scores of all the students after each test were ranked based on the student's performance and allowed us to classify the upper one-third of students as high achievers and the lower one-third as low achievers [16,17].

Difficulty Index (DIF)

Calculated using the following formula: H + L/N.

H = Number of students that correctly answer the item in the high-achieving group

L = Number of students that correctly answer the item in the lower achieving group

N = Total number of students in the two groups

If the DIF is less than 30%, the item is considered very difficult; if it is more than 70 %, it is considered easy. However, the DIF ranging from 30 % to 70 % was taken as an acceptable range. The DIF and DI are reciprocally related.

Discrimination index (DI)

They were calculated using the formula DI = H - L /N*2 [18].

Where the DI is:

Negative items have poor discrimination.

0.19 or less, the item has poor discrimination.

Between 0.2 and 0.29, the item has acceptable discrimination.

Between 0.3 and 0.9, the item has good discrimination.

Greater than equal to 0.4, the item has excellent discrimination.

The DI measures the differences obtained in correct responses between the higher achieving and the lower achieving group and has a value that ranges between 0 and 1. The higher the DI, the more the test item can discriminate better between students with higher and lower test scores. These items were analyzed using the point-biserial correlation. In a point-biserial correlation, test scores on a continuous scale are compared to a single item with only two values, which are either correct or incorrect. The point-biserial correlation is the correlation between two options: the right or the wrong scores that students receive on a given item and the total scores that the students receive when summing up their scores across the remaining other items. It is a particular type of correlation between a dichotomous variable (the multiple-choice item score, which is right or wrong, 0 or 1) and a continuous variable.

Distracter Efficiency (DE)

The distractors are measures of item functioning. When a distracter is chosen by >5% of participants, it is considered a functioning distracter (FD), and if chosen by <5% of participants is a non-functioning distractor (NFD). When an item does not have an NFD, it has a value of 99.9%; however, items with three, two, or one NFDs have a DE of 99.9%, 66.6%, and 33.3%, respectively [13].

## Results

One hundred eighty-nine students were examined using 300 MCQ items in five different test sessions. The student scores ranged from 28.3 to 90.0%, with a mean score of 64.6 ±4.35. The 300 MCQ items have 100 distractors, with a KR reliability index ranging from 0.744 to 0.831 across the five tests (Table 1).

**Table 1:** Psychometric Measurements The mean test score per test was 64.6%±4.35 The mean reliability across the five tests was 0.793±0.039

Measurement	2023	2022	2021	2020	2019	Total
No of items (N)	60	60	60	60	60	300
No of examinees (N)	41	39	36	25	48	189
Difficult MCQs (N)	1	3	4	6	6	20
Easy MCQs (N)	7	10	6	15	16	54
Acceptable MCQs (N)	52	47	50	39	38	226
Mean test score (%)	70.63	63.72	62.07	59.54	67.17	NA/(-)
Range of test score (%)	35-90	28.3-83.3	36.7-83.3	30.2-81.1	41.7-76.7	NA/(-)
KR-20 reliability	0.831	0.744	0.817	0.817	0.760	NA/(-)

Difficulty index (DIF)

Questions with scores of less than 30.0% responses were considered as difficult. The total number of difficult questions in the 300-item MCQ was 20 (6.7%), ranging from one to six questions in every 60-item test administered. The mean number of difficult questions was 4± 2.121 (95% CI =1.37-6.63) per session. In the 300-item MCQ assessed, 54 (18%) of the questions were easy (scores >70%) with a mean of 10.8±4.55 (95% CI= 5.15-16.45). The acceptable MCQs (scores of 30-70%) were 226 (75.3%) with a mean of 45.2±6.38 (95% CI =37.28-53.12) in the 300-item MCQ assessed over the five years (Table 1).

DIs

The mean and SD discrimination indices are shown in Table 2. There were 110/300 (36.7%) items with poor discrimination services (point-biserial coefficient ≤0.19). The mean number of questions with poor discrimination in the 300 test items was 22±6.519. There were 190 questions with good discrimination (63.3%). Good discrimination questions ranged from 27 to 44 across the 60-item tests with a mean of 38±6.519 per session. Of these 190 questions, 69 (23%) had an acceptable level of discrimination (point-biserial coefficient 0.2-0.29), 66 (22%) had good discrimination (point-biserial 0.3-0.39), and 55(18.3%) of the questions had excellent (point-biserial coefficient ≥0.4) in discriminating between higher and lower achievers (Table 3). The results show no question with a negative point-biserial coefficient.

**Table 2:** Item Analysis of Surgery Course Examinations 2019-2023 Significant P-value <0.05 Not Significant P-value >0.05 FD: functional distractors; NA/(-): not applicable

				95% Confidence Interval		
Item Analysis						
	Mean/test	SD	t-test	Lower	Upper	P-value
Item features						
Difficult MCQs	4	2.12	4.22	1.37	6.63	0.014
Easy MCQs	10.8	4.55	5.31	5.15	16.45	0.006
Difficulty index	45.2	6.38	15.84	37.28	53.12	NA/(-)
Bi-serial correlation						
Point-biserial ≤0.19	22	6.52	7.55	13.91	30.09	0.002
Point-biserial 0.2-0.29	13.8	3.96	7.79	8.88	18.72	0.001
Point-biserial 0.3-0.39	13.2	6.06	4.87	5.68	20.72	0.008
Point-biserial ≥0.4	11	6.59	3.73	2.81	19.19	0.02
Distractors						
FD per item	2.19	0.101	48.60	2.06	2.32	NA/(-)
FD per test	133.8	6.18	48.40	126.13	141.47	NA/(-)
3 FD	20.4	3.05	14.96	16.61	24.19	
2 FD	31	2.83	24.51	27.49	34.51	
1 FD	8.6	2.88	6.68	5.02	12.18	0.003
KR-20	0.793	0.04	45.51	0.75	0.84	NA/(-)
Discrimination						
Good ≥0.2	38	6.52	13.03	29.91	46.09	NA/(-)
Frequency <5% +Poor Discrimination ≤0.19	68.2	12.39	12.30	52.81	83.59	NA/(-)

**Table 3:** Distractors' Efficiency Significant P-value <0.05 Not Significant P-value >0.05 NA/(-): not applicable

Performance	2023	2022	2021	2020	2019	Total
Number of items	60	60	60	60	60	300
No of the distractors assessed	180	180	180	180	180	900
MCQ with						
Poor discrimination ≤0.19	19	33	22	20	16	110
Good discrimination ≥ 0.2	41	27	38	40	44	190
Functioning distracter per test (N=180)	140(77.8%)	124(68.9%)	132(73.3%)	137(76.1%)	136(75.6%)	669
Functioning distracter per item (n %)						
Three	21(35%)	16(26.7%)	19(31.7%)	22(36.7%)	24(40.0%)	102
Two	28(46.7%)	32(53.3%)	34(56.7%)	33(55.0%)	28(46.7%)	155
One	11(18.3%)	12(20%)	7(11.7%)	5(8.3%)	8(13.3%)	43
Mean Functioning Distractor per item	2.17	2.03	2.2	2.28	2.27	NA/(-)

Distracter efficiency (DE)

There were 900 distractors in the 300 items analyzed. All the distractors were functional (FD), and none were observed to be non-functional. The number of functional distractors per test item ranged from 1 to 3. The mean functional distracter per test item was 2.19±1.007. Similarly, the mean number of items with three FDs was 20.4±3.05, two FDs 8.6±2.88, and one FD was 2.19±0.101. Of the 300 items analyzed, 102 (34%) had three FDs, 51.7% had two, and 43(14.3%) had one functional distractor. The DE for the 300-item level exam ranged between 50 and 100%. Questions with three FDs had 99.9% distractor efficiency, while those with two FDs and one FD had 66.6% and 33.3% distractor efficiency, respectively.

## Discussion

The assessments are significant for both students and educators. For students, this assessment serves as a measure of their knowledge and competence in surgery. It allows them to identify areas of strength and weakness, helping them focus on improving their understanding of specific topics or skills. Additionally, the assessment also serves as a motivating factor for students. For educators, the test assessment provides valuable feedback on their teaching methods and curriculum effectiveness.

Preparations and writing MCQs is a challenging task, often time-consuming, especially among the newly appointed faculty. It has become imperative from time to time to evaluate MCQ items to see how effective they are in assessing students' knowledge [19]. In adopting an appropriate assessment strategy, MCQ is the most used format in assessing the student's knowledge as part of curriculum development. It is vital to evaluate MCQ items to see their efficiency in assessing students' knowledge [20]. In integrated competency-based medical education programs, consideration is given to the quality of student assessment. Assessments that have high validity and reliability are known to be precursors of learning activities and promote competence [20].

Findings demonstrated that the psychometric properties of the items were good. Although there were some identified gaps, the quality of the exam administered can support sound decision-making. Nevertheless, two-thirds of the items in this study had good DIs (Table 2). We have an excellent DIF, reliability of items, and full functioning of distractors. Encouraging mean item discrimination within an acceptable range encourages us to be confident in the validity of the decisions. Most of the questions (75.3%) are within the acceptable range of the DIF. This study's mean item DIF was determined to be 45.2±6.38 (95% CI 37.28%-53.12%), which is in the desirable range (30-70%) [21,22]. In our study, 6.7% of the questions were difficult but similar to what was reported by other studies elsewhere (2-19%) [23]. The easy questions constituted 18% of the items examined. However, this finding on easy questions is similar to what was reported by Patil (18%), higher than what was reported by Rao (13.5%) [21]. Easy questions have been advocated by many to either be removed or the items could be placed as early questions to stimulate the examination process.

The proportion of items with a DI of 0.2 and above was 63.3%, and items with such discrimination levels are accepted for reuse. This level of eligible questions for reuse is similar to the level reported by Balay in Ethiopia (22.3%) and far less than what Rao reported from India (85%) [21,24]. The study showed that about one-third (36.7%) of the items have a poor discriminatory index (<0.19). Many reasons have been considered responsible for getting poor DI, like using wrong keys, ambiguity in the stem, areas where opinion differs, and often controversial. A subject expert should revise defective items to improve the standards of these items. Such items can be reconstructed, and feedback should be sent to the faculty for re-evaluation. It is not advisable to drop defective content because some skills may be included in the assessment [25]. In this study, items were reviewed for technical flaws; our program has a competitive process under a highly qualified student assessment committee. All our examination questions must undergo review by the committee before approval, but no external item review was done before the administration of the exam questions.

One of the tasks identified in creating quality MCQs is having efficient distractors as options. All the distractors used in these tests were functional, giving 100% DE. Elsewhere studies have shown that only about 7-20% of MCQs had no functioning distractors, which was entirely different from what this study found [26]. The level of DE seen here has surpassed the reported levels of DE in India (85%) [21]. This study reports the KR20 index of the tests in the range of 0.793-0.844, indicating acceptable reliability and agreed with other studies [27].

The assessment implies that after analyzing students' performance, educators can identify areas where students struggle and modify their teaching strategies accordingly. This assessment also helps identify gaps in the curriculum, ensuring that all essential topics are adequately covered. Furthermore, the assessment results can be used for program evaluation and accreditation purposes, helping educators maintain the quality of surgical education.

Limitations of the study: This study has limitations in getting generalized because only one course was analyzed. However, it was an analysis of five different tests, each with 60 items, over 5 years. It would be more robust and precise if many courses were pooled together and analyzed, that could show a trend of psychometric properties across various courses and provide learning opportunities among medical educators.

## Conclusions

The psychometric indices used in assessing these tests have been encouraging, with the DIF, distracter efficiencies, and item reliability within acceptable standards. We recommend robust faculty training and capacity-building to improve the item writing skills of our staff. Additionally, the study's findings suggest that the faculty requires feedback, especially on items with shallow discrimination values. This will involve revising items by the content experts and determining the suitability and validity of the questions before conducting any test or assessment.
